# A 5-Year Retrospective Review of Fungal Keratitis at Hospital Universiti Sains Malaysia

**DOI:** 10.1155/2012/851563

**Published:** 2012-12-16

**Authors:** Fadzillah Mohd-Tahir, A. Norhayati, Ishak Siti-Raihan, M. Ibrahim

**Affiliations:** Department of Ophthalmology, School of Medical Sciences, Universiti Sains Malaysia, Kelantan, 16150 Kubang Kerian, Malaysia

## Abstract

*Background*. Corneal blindness from healed infected keratitis is one of the most preventable causes of monocular blindness in developing countries, including Malaysia. Our objectives were to identify the causative fungi, predisposing risk factors, the proportion of correct clinical diagnosis, and visual outcome of patients treated in our hospital. *Methods*. A retrospective review of medical and microbiology records was conducted for all patients who were treated for fungal keratitis at Hospital Universiti Sains Malaysia from January 2007 until December 2011. *Results*. Forty-seven patients (47/186, 25.27%) were treated for fungal keratitis during the study period. This demonstrated that the incidence of fungal keratitis has increased each year from 2007 to 2011 by 12.50%, 17.65%, 21.21%, 26.83%, and 28.57%, respectively. The most common predisposing factors were injury to the eye followed by use of topical steroid, and preexisting ocular surface disease. *Fusarium* species were the most common fungal isolated, followed by *Candida* species. Clinical diagnosis of fungal keratitis was made in 26 of the 41 (63.41%) cases of positive isolates. Of these, in eleven cases (23.40%) patients required surgical intervention. Clinical outcome of healed scar was achieved in 34 (72.34%) cases. *Conclusions*. The percentage of positive fungal isolated has steadily increased and the trend of common fungal isolated has changed. The latest review regarding fungal keratitis is important for us to improve patients' outcome in the future.

## 1. Introductions 

Corneal blindness is the second most common cause of blindness, after cataract, in developing countries. The World Health Organisation estimated that in every year, about 1.5–2.0 million new cases of monocular blindness in developing countries is secondary to corneal ulceration [1]. Among infectious corneal ulcers, fungal keratitis is the most challenging and yet most commonly found in agricultural or developing countries including Malaysia. From the year 2008, Hospital Universiti Sains Malaysia's Corneal Unit has been the main referral centre for problematic cases of corneal disease for the East Coast of Peninsular Malaysia. There are three states along the East Coast of Peninsular Malaysia, which covers an area of 63846 km^2^ and an estimated population of 3.8 million people in 2007. We reported the causative fungi, risk factors, and outcome of all patients treated from fungal keratitis in our hospital over the past five years. 

Although previous studies were conducted on infective keratitis in Malaysia [2–4], our study is the first study concentrated on fungal keratitis in order to better quantify and qualify its characteristics. Furthermore, our study covered a large period of 5 years compared to the 1 year study period by Norina et al. [2] and Kursiah et al. [3]. However, this study revealed similar results where the predominant etiological agent causing fungi keratitis was *Fusarium* sp.

## 2. Materials and Methods 

We conducted a retrospective review of all medical and microbiology records for all cases treated with fungal keratitis at Hospital Universiti Sains Malaysia from January 2007 until December 2011. An analysis was performed to study the demographic features, possible predisposing factors, duration of symptoms, microbiology results, therapy received, and visual outcome at the end of three months or at the completion of the treatment (whichever was earlier). 

Following clinical diagnosis of infective corneal ulcer based on slit-lamp biomicroscopic examination, the patient's corneal were scraped and sent for microbiological investigations as per institutional' protocol. Using standard techniques, corneal scraping was performed to all corneal ulcer patients under aseptic condition using 21 gauge needles or a Kimura spatula following the instillation of local anaesthesia (4% proparacaine eye drops). The material collected from the leading edge and base of the ulcer was inoculated directly onto blood agar, chocolate agar, McConkey agar for bacteria, and Sabaroud's agar for fungal culture. Two smears were made onto two slides. One slide was stained with Gram stain and the other with 10% KOH preparation for direct microscopic examination [2, 5–7]. For all cases, empirical treatment was given while waiting for the microbiological test and was later changed according to the results or the patients' responses. Pathogen isolates were defined as any positive result in gram stains or culture agar. The surgical mode of treatment included tissue adhesive application with bandage contact lenses or tissue patches, penetrating keratoplasty and evisceration, whenever applicable. The treatment outcome was analysed in terms of healed scars and visual acuity. One patient was excluded from the study due to incomplete records. 

## 3. Results 

**Figure 1:**
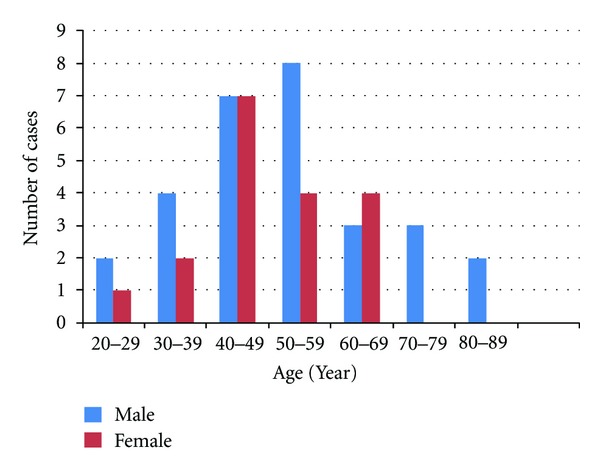
Age and sex distributions of patients with fungal keratitis at Hospital Universiti Sains Malaysia from 2007 until 2011.

A total of 186 patients were diagnosed with infective keratitis from January 2007 until December 2011. Forty-seven patients (25.27%) were treated with fungal keratitis. Demographic data was summarised in Figure 1. Males were more common than the females. The majority of patients (65.96%) were aged between 30 to 60 years old. Two thirds of the fungal keratitis cases (68.29%) were presented to us within a week of the onset of their symptoms. During the first presentation, about half of the cases (53.66%) had a visual acuity of a counting finger or worse, while nearly one third (29.27%) had a visual acuity of more than 6/18. Five of our patients suffering from fungal keratitis (10.6%, 5/47) were diagnosed with diabetes mellitus only during this screening process. All of them had good control of their random capillary blood sugar (less than 11 mmol/L) throughout their stay in hospital.

The most common predisposing factors for developing fungal keratitis in our patients was injury to the eye (23/47, 48.94%) followed by use of topical steroids (8/47, 17.02%) and preexisting ocular surface disease (5/47, 10.64%). Two patients (4.26%) wore contact lenses for their recurrent epithelial erosions. One third of the patients who had injuries with vegetative material were directly related to agricultural work at rubber tree estates. 

**Figure 2:**
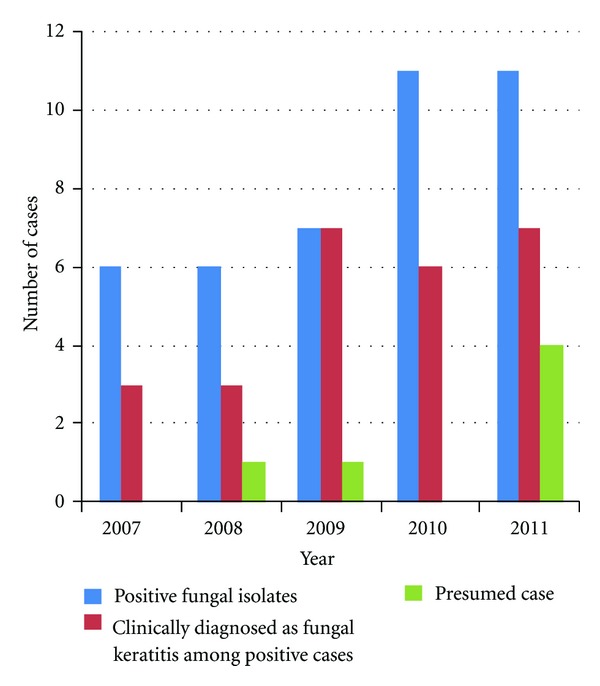
Comparison between numbers of positive fungal isolates, correct clinical diagnosis, and presumed cases of fungal keratitis.

In practise, we usually have a low threshold of suspecting that fungal is a causative agent for keratitis even with no history of trauma by vegetative material in a warm climate area like Malaysia where the domestic source of fungi cannot be excluded. Therefore, corneal scraping will include a culture in Sabaroud agar for fungal culture and will also be slide stained with 10% KOH preparation for direct microscopic examination. For all cases, empirical treatment was given while waiting for the microbiological test and will be changed later according to the results in all our keratitis patients. Forty-one (41/186, 22.04%) cases of all infective corneal ulcers had isolated fungal with five (5/41, 12.20%) of them suffering from bacterial coinfection and one case (1/41, 2.44%) suffered from polyfungal infection. Six (3.23%) cases were presumed to be suffering from fungal keratitis with two of them presuming to be suffering from bacterial coinfection. *Fusarium* species (46.34%, 19/41) were the most common fungal isolated, followed by *Candida* species (12.20%, 5/41). Clinical diagnosis of fungal keratitis was made in 26 out of 41 (63.41%) cases of positive isolates. The type of fungal isolated and comparisons made between clinical diagnosis and positive fungal isolated was summarised in Table 1 and Figure 2. 

Eight out of the positive fungal isolates patients (19.51%, 8/41) required therapeutic penetrating keratoplasty and two of the patients required prolonged topical steroid for previous graft. Two (4.88%, 2/41) of the positive fungal isolates patients had a corneal patch graft. One of the presumed cases required a tectonic penetrating keratoplasty. Two patients (4.26%, 2/47) required evisceration, although one of them refused. 6.38% (3/47) received glue application with bandaged contact lenses (BCL) for impending or small perforations. The treatment modalities are summarised in Table 2. The clinical outcomes of healed scars was achieved in 34 (72.34%, 34/47) cases. At the end of the study, 22 (46.81%) of patients had visual acuity better than 6/18, 20 (42.55%) had visual acuity of a counting finger, or worse with 5 (10.64%) of them absolutely losing their vision. When analysing each individual case, 25 (53.19%) of patients had improved visual acuity, 11 (23.40%) unchanged, and 11 (23.40%) worsened compared to visual acuity at presentation. All of the patients completed a minimum of six-week duration period of treatment but subsequently, 29.795% (14/47) were lost during follow-up. 

## 4. Discussion 

The prevalence of fungal keratitis in our hospital was 25.27% and comparable with our neighbouring countries of Singapore and Thailand [8, 9]. Our gender predilection and age distributions were also similar. In our study, the majority of subjects were middle-aged men involved in agricultural work. Trauma was the leading predisposing factor while the wearing of contact lenses was least commonly related to the fungal keratitis. One third of the cases had injury with vegetative material and directly related to their field of work in rubber tree estates and only two of the cases involved suffered injury due to household material, in contrast to Singapore where one third of their cases were related to material at construction sites [8]. We also had three cases with a history of foreign bodies from animals. These explained that in our region, the great variety of sources of fungal exist and are not confined to plantation and agricultural only. 

Other developing countries reported that *Fusarium* species were the most commonly isolated fungal followed by the *Aspergillus* species [10–13]. Reports from China showed an increasing number of *Fusarium* species but decreasing numbers of *Aspergillus* species isolated over the past decade [11]. *Fusarium* species remained the most commonly isolated fungal in our keratitis patients. Previously in the year 2004, the *Aspergillus* species were the second most common fungal isolated in our hospital [2]. However, the trend has changed where *Candida* species become the second most common isolated fungal. This is consistent with reports from Philadelphia where fungal keratitis due to *Candida* species was more common than due to the *Aspergillus* species [14]. Corticosteroid use and history of corneal transplants have been reported to be fundamental risk factors for developing *Candida parapsilosis* keratitis [15]. Our result is consistent with this where only *Candida Parapsilosis* was isolated from the corneal graft of two patients who were also on topical steroids. Corneal graft has become more frequently used as an indicative method for several signs in our hospital since the establishment of our Corneal Unit. There might possibly be more *Candida parapsilosis* keratitis in the future as the number of patients with corneal graft increases. Although Pate et al. [16] reported that bacteria coinfection was three times more commonly found in yeast keratitis than with filamentous fungal keratitis, none of our yeast keratitis has bacterial coinfection. We observed that the *Fusarium* species appear to be the most common fungal keratitis and have either bacterial coinfection or polyfungal infection. 

In our study, the number of patients treated with fungal keratitis fluctuated over the past 5 years. However, the percentage of fungal isolated steadily increases each year (12.50%, 17.65%, 21.21%, 26.83%, 28.57%). In 2003, there were 17% reported fungal corneal ulcers in Ipoh Malaysia [3]. While in 2004, the percentage of fungal ulcers isolated was only 14% at our institution from a study initiated by Norina et al. [2]. The increase in the trend of isolated fungal culture may reflect the improvement of scraping techniques carried out and also the formation of the Corneal Unit at Hospital Universiti Sains Malaysia in the year 2008. Our referral cases for microbial corneal ulcer within this 5-year period were increased to around 187 of cases. This is considered a high number, compared to Singapore, which had only 29 cases from 1991 to 1995 [8]. Treatment may heavily rely on clinical diagnosis and the patient's clinical response to the antifungal prescribed. Clinical diagnosis of fungal keratitis was made in 26 out of 41 (63.41%) cases of positive fungal isolates. This is slightly lower than the study done in Eastern India [17] where 70.5% of their positive fungal cultures were clinically diagnosed as fungal keratitis. However, our rate of positive fungal isolates among clinically diagnosed patients had increased from 42.86% in 2007 to 63.64% in 2011 with an average of 5.20% positive cases each year. Clinical diagnosis of fungal keratitis can be problematic especially when there occurred a delay in presentation or multiple medications prior to referral to tertiary eye care. 

Overall, the difficulties in treating fungal keratitis in our setting are due to late presentation, delay and insufficiency of lab confirmatory, delay in the initiation of antifungal due to lack of ability in making clinical diagnosis, limited types of antifungal available, and resistance to certain antifungal. We observed four (4/41, 9.76%) cases of keratitis due to *Aspergillus* species, three cases of nonsporulatingmolds (3/41, 7.32%), and five unidentified genera (5/41, 12.20%) during the study period. In Chennai, 12% of the keratitis was due to nonsporulatingmold with 54% of them being newly emerging pathogens based on PCR [18]. If this result applied to us, we have to improve our eye care system in order to deal with this new emerging fungus. We also have very limited sources of corneal donors. Nevertheless, our clinical outcome of healed scaring was achieved in 34 (72.34%, 34/47) cases. At the end of the treatment, more than half of the cases had improved visual acuity, a quarter unchanged, and less than a quarter had worsened visual acuity. 

Our positive isolated fungal ulcer increased in trend from 12.5% in 2007 to 28.5% in 2011. However, this is quite low in number compared to South India where the increase in trend was up to 78% in 2009 [19]. The geographical variation and economic factors may play a vital role in this wide variation of percentages. In temperate climates, such as in Britain and northern United States, the incidence remains low [20, 21].

## 5. Conclusions 

The percentage of positive fungal isolated has steadily increased in our institution with a mean of 4.02% each year. The trend of common fungal isolated has changed. The latest review of all clinical and laboratory aspects regarding fungal keratitis is important for us in order to formulate better diagnosis and treatment strategies, hence to improve patients' outcome in the future. Since agriculture sectors contributing to the development of our country and fungal infection are common, improvement in our eye care systems is crucial in preventing blindness in our region.

## Figures and Tables

**Table 1:** Type of fungal ulcer from corneal scrapping.

Type of fungal	No of cases (%) (*n* = 41)
Hyaline	
*Fusarium* sp.	19 (46%)
*Aspergillus* sp.	4 (9.75%)
*Scedosporium apiospermum *	1 (2.44%)
*Trichoderma *sp.	1 (2.44%)
*Epidermophyton floccosum *	1 (2.44%)
Yeast	
*Candida albicans *	2 (4.87%)
*Candida parapsilosis *	2 (4.87%)
*Candida tropicalis *	1 (2.44%)
Dematiaceous	
*Curvularia* sp.	2 (4.87%)
Nonsporulating fungi	3 (7.31%)
Unidentified hyaline	4 (9.75%)
Unidentified yeast	1 (2.44%)

**Table 2:** Treatment modalities and outcome according to each fungal isolates.

Type of fungal (*n*)	Vision at presentation					Vision posttreatment			
	<6/18	6/18–1/60	CF-PL	NPL	Treatment	less than 6/18	6/18–1/60	CF-PL	NPL
Hyaline									
*Fusarium *sp. (19)	6	4	9		G. Natamycin if available, G. Amphotericin. B, Oral & Gut Fluconazole. 5 PK, 1 evisceration	10	3	4	2
*Aspergillus *sp. (4)		1	3		G. Natamycin, Voriconazole, Amphotericin B Oral Fluconazole. 1 scleral patch, 2 PK			3	1
*Scedosporium* sp. (1)			1		Gutt Amphotericin B & Voriconazole. Oral Voriconazole.	1			
*Trichoderma* sp. (1)	1				Gutt. Ciprofloxacin (Provisional diagnosis: marginal keratitis)	1			
*Epidermophyton *sp. (1)	1				Gutt. Amphotericin B & Fluconazole. Oral Fluconazole.	1			

Yeast									
*Candida albicans* (2) (1 immunocompromised patient with bilateral involvement)	1		1		Gutt Amphotericin B	1		1	
*Candida parapsilosis* (2)		1	1		Gutt Amphotericin B		1	1	
(infected cornea graft)					Repeat penetrating keratoplasty				
*Candida tropicalis* (1)				1	Gutt Amphotericin B				1
(Ocular ischaemic syndrome)									

Dermatiaceous									
*Curvularia* sp. (2)	2				Gutt. Amphotericin B	2			

Nonsporulating fungi (3)	1		2		Gutt. Amphotericin B	2	1		

Unidentified hyaline (4)	1		3		All required systemic + topical treatment Natamycin in one case. One required PK.	2		2	

Unidentified yeast (1)			1		Gutt Amphotericin B		1		

Abbreviations: CF: counting finger, PK: penetrating keratoplasty, NPL: no perception of light, PL: perception of light.
